# CRM1/XPO1 is associated with clinical outcome in glioma and represents a therapeutic target by perturbing multiple core pathways

**DOI:** 10.1186/s13045-016-0338-2

**Published:** 2016-10-12

**Authors:** Xuejiao Liu, Yulong Chong, Yiming Tu, Ning Liu, Chenglong Yue, Zhenglei Qi, Huize Liu, Yao Yao, Hongmei Liu, Shangfeng Gao, Mingshan Niu, Rutong Yu

**Affiliations:** 1 Insititute of Nervous System Diseases, Xuzhou Medical University, Xuzhou, Jiangsu China; 2 Brain Hospital, the Affiliated Hospital of Xuzhou Medical University, Xuzhou, Jiangsu China; 3 Jiangsu Key Laboratory of Bone Marrow Stem Cell, Blood Diseases Institute, Xuzhou Medical University, Xuzhou, Jiangsu China; 4 Nanjing Durm Tower Hospital Group, Suqian City People’s Hospital, Suqian, Jiangsu China

**Keywords:** Glioma, CRM1 inhibitor, S109, Proliferation, Cell cycle

## Abstract

**Background:**

Malignant gliomas are associated with a high mortality rate, and effective treatment options are limited. Thus, the development of novel targeted treatments to battle this deadly disease is imperative.

**Methods:**

In this study, we investigated the in vitro effects of the novel reversible chromosomal region maintenance 1 (CRM1) inhibitor S109 on cell proliferation in human gliomas. S109 was also evaluated in an intracranial glioblastoma xenograft model.

**Results:**

We found that high expression of *CRM1* in glioma is a predictor of short overall survival and poor patient outcome. Our data demonstrate that S109 significantly inhibits the proliferation of human glioma cells by inducing cell cycle arrest at the G1 phase. Notably, we observed that high-grade glioma cells are more sensitive to S109 treatment compared with low-grade glioma cells. In an intracranial mouse model, S109 significantly prolonged the survival of tumor-bearing animals without causing any obvious toxicity. Mechanistically, S109 treatment simultaneously perturbed the three core pathways (the RTK/AKT/Foxos signaling pathway and the p53 and Rb1 tumor-suppressor pathways) implicated in human glioma cells by promoting the nuclear retention of multiple tumor-suppressor proteins.

**Conclusions:**

Taken together, our study highlights the potential role of CRM1 as an attractive molecular target for the treatment of human glioma and indicates that CRM1 inhibition by S109 might represent a novel treatment approach.

**Electronic supplementary material:**

The online version of this article (doi:10.1186/s13045-016-0338-2) contains supplementary material, which is available to authorized users.

## Background

Gliomas account for approximately 70 % of all primary malignant brain tumors [1]. On the basis of histological features, gliomas are classified into four distinct subtypes by the World Health Organization [2]: grade I (pilocytic astrocytoma), grade II (diffuse astrocytoma), grade III (anaplastic astrocytoma), and grade IV (glioblastoma). Glioblastomas account for approximately 60 to 70 % of malignant gliomas. Despite advances in the understanding of glioma biology and the development of new treatment options, the median survival for patients with glioblastomas is merely 12 to 15 months [3]. Thus, there is an urgent need for the development of novel targeted therapeutics.

Two decades of molecular studies together with recent large-scale cancer gene sequencing efforts have identified three critical signaling pathways that are misregulated in human glioblastomas: the RTK/PI3K/AKT/Foxos signaling pathway and the p53 and Rb1 tumor suppressor-pathways [4–6]. Consistent with these findings, the simultaneous deletion of *Pten*-, *p53*-, and *Nf1*-mediated CRISPR/Cas9 can promote the development of highly aggressive tumors resembling human glioblastomas in the mouse brain [7]. Furthermore, nearly all subtypes of gliomas are associated with the amplification and overexpression of the epidermal growth factor receptor (EGFR) gene [8]. However, inhibitors of receptor tyrosine kinases (RTK) [9–11], PI3K [12], and mTOR [13, 14] have exhibited only modest activity in glioma, with response rates of 0 to 15 % and no prolongation of 6-month progression-free survival [15, 16]. Given the complexity and redundancy of the signaling networks associated with glioma, the simultaneous targeting of critical oncogenic pathways might constitute a promising treatment approach.

Chromosomal region maintenance 1 (CRM1), also referred to as exportin 1 (XPO1) [17], is a promising therapeutic target for gliomas. Increased CRM1 expression has been observed in gliomas and is correlated with a poor prognosis and higher grade of malignancy [18]. CRM1 is a key member of the karyopherin β superfamily of nuclear transport receptors, which mediate the transport of specific proteins from the nucleus to the cytoplasm in eukaryotic cells [19]. CRM1 is the key exporter of multiple tumor-suppressor proteins [20], including Foxos, Rb1, p53, p21, p27, and survivin. Accumulating lines of evidence suggest that the misregulation of nuclear protein export dynamics is involved in cancer cell survival, tumor progression, and drug resistance [21, 22]. These observations have stimulated considerable interest in drugs targeting the nuclear export of proteins.

Leptomycin B (LMB), the first natural inhibitor of CRM1 to be identified, can covalently bind the Cys528 residue in the cargo-binding region of CRM1 [23]. However, the phase I clinical trial of LMB was terminated due to its toxic effects and lack of efficacy at tolerable doses [24]. Recently, a novel class of selective inhibitors of nuclear export (SINE) has been developed [25–27]. One member of this class, selinexor (KPT-330), is currently undergoing phase I/II clinical trials to evaluate its effect in several solid and hematologic malignancies [28]. KPT-330 is a CRM1 inhibitor that forms a slowly reversible covalent bond with CRM1, and preliminary evidence indicates that it exhibits a relatively favorable drug tolerability profile. These findings suggest that a selective and reversible inhibitor might offer an improved tolerability profile. However, most of the currently available CRM1 inhibitors function by irreversibly binding to Cys528. Recently, we developed the novel reversible CRM1 inhibitor S109, which can induce CRM1 protein degradation [29].

In the present study, we evaluated the mechanism and therapeutic potential of a reversible CRM1 inhibitor, S109, in the treatment of human glioma. Specifically, we investigated the therapeutic efficacy of S109 in vitro and in intracranial mouse models of malignant glioma and elucidated the mechanism underlying S109-mediated anti-glioma activity. This study provides a basis for further clinical investigations of S109.

## Methods

### Glioma and non-tumor human brain tissues

Human glioma specimens (obtained through surgical resection) and non-tumorous brain tissues (obtained from patients with internal decompression in cerebral trauma) were obtained from the Affiliated Hospital of Xuzhou Medical College (Xuzhou, China). Written informed consent was obtained from all of the participants, and this study was approved by the Ethics Committee of the Affiliated Hospital of Xuzhou Medical University.

### Cell lines and culture conditions

The human glioma cell lines U251 and SHG-44 and glioblastoma cell lines U118 and U87 were purchased from the Shanghai Cell Bank, Chinese Academy of Sciences. All of the cell lines were cultured in Dulbecco’s modified Eagle’s medium (DMEM) (Gibco, Carlsbad, CA, USA) supplemented with 10 % fetal bovine serum (FBS, Gibco) in a humidified incubator with 5 % CO_2_ at 37 °C.

### Antibodies and reagents

Primary antibodies against CRM1, GAPDH, actin, RanBP1, and p53 were obtained from Santa Cruz Biotechnology (Santa Cruz, CA, USA), and antibodies against cyclin D1, Cdc25B, p27, p21, Foxo1, p-Foxo1, Akt, p-Akt, p-Rb1, and histone H3 were purchased from Cell Signaling Technology (CST, Beverly, MA, USA).

### Cytotoxicity assay

The cell counting kit-8 (CCK-8) assay was used to assess cell viability following S109 treatment. Briefly, glioma cells were plated in triplicate in 96-well plates (2 × 10^3^ cells per well). After overnight incubation, the cells were treated with various concentrations of S109 for 72 h. The cells were subsequently washed three times with phosphate-buffered saline (PBS), and 10 μL of CCK8 was added to each well. Following a 3-h incubation, the absorbance was measured using a microplate reader at a wavelength of 450 nm.

### EdU incorporation assays

Cell proliferation was assessed using the Cell-Light™ EdU Cell Proliferation Detection Kit (RiboBio, China) according to the manufacturer’s instructions. The cells were treated with various concentrations of S109 for 12 h. Subsequently, the cells were incubated with 50 μM EdU for 2 h and then fixed in 4 % paraformaldehyde for 30 min. After permeabilization with 0.5 % Triton X-100, the cells were incubated with Apollo® reaction cocktail for 30 min in the dark. The cellular DNA was stained with DAPI for 15 min. Following three washes with PBS, the cells were examined and imaged using an inverted microscope (Olympus, Japan).

### Colony formation assay

The cells were seeded at a density of 500 cells/well in 6-well culture plates. The plated cells were treated with different concentrations of S109 for 12 h, and fresh medium was subsequently added. After a 14-day incubation, the cells were fixed in methanol for 15 min and stained with 0.1 % crystal violet solution. Positive colony formation, defined as colonies with more than 50 cells, was confirmed by manual counting.

### Co-immunoprecipitation

Immunoprecipitation assays were performed as previously described [30]. Briefly, cells treated with 0.1 % DMSO or S109 were lysed in cold lysis buffer. The supernatant was incubated with anti-CRM1 antibody for 8 h and then with protein G-Sepharose 4B (Roche, Basel, Switzerland) overnight at 4 °C while rocking. The immunoprecipitated complexes were washed three times with lysis buffer and analyzed by western blot.

### Immunofluorescence microscopy

The cells were then treated with S109 and subsequently fixed for 20 min with 4 % paraformaldehyde in PBS. The cell membranes were subsequently permeabilized in 0.1 % Triton X-100 and blocked with 1 % bovine serum albumin (BSA) in PBS. The cells were then incubated with the indicated antibody. DAPI was used for nuclei labeling (blue), and the stained cells were visualized and imaged through fluorescence microscopy (Olympus, Japan).

### Establishment of CRM1-WT and CRM1-C528S stable cell lines

The cDNA encoding human CRM1-WT or CRM1 with the C528S mutation was inserted into the pWPXLd-puro lentiviral vector containing a sequence encoding a flag tag. The viruses were produced in 293FT cells by co-transfecting the recombinant plasmids with the helper plasmids pSPXA2 and pMD2.G. The U87 cells were then transfected with the CRM1-WT or CRM1-C528S lentivirus for 48 h and then continuously cultured in medium containing 2.5 μg/mL puromycin. The surviving cells were cultured and used to generate cell lines that stably expressed CRM1-WT or CRM1-C528S.

### CRM1 expression and survival analysis in patients with glioma

CRM1 gene expression datasets were obtained from R2: microarray analysis and visualization platform (http://hgserver1.amc.nl/cgi-bin/r2/main.cgi). The prognosis analysis was conducted online, and cutoff values for separating high and low expression groups were determine by auto scan.

### In vivo studies

All animal experimental protocols were approved by the Ethics Committee of the Xuzhou Medical University. Male athymic BALB/c nude mice aged 5 to 6 weeks were obtained from the Experimental Animal Center of Xuzhou Medical College. Firefly luciferase-labeled U87 cells (5 × 10^5^ cells per mouse) were intracranially injected into the right striatum of nude mice using a small animal stereotactic apparatus as described in our previous report [31, 32]. Once the presence of a tumor was confirmed by imaging system, the tumor-bearing mice were randomly divided into one of the following three treatment groups (*n* = 8 per group): S109 at 20 mg/kg, S109 at 50 mg/kg, and vehicle. The drugs and vehicle were delivered daily via intraperitoneal injections. Tumor growth was monitored at regular intervals by injecting D-luciferin 10 min prior to imaging using a NightOWL LB 983 small-animal in vivo imaging system (Berthold Technologies, Germany).

### Histopathology and immunofluorescence staining

The whole brain and vital organs (lung, liver, testis, kidney, and heart) of the control and treated mice were harvested on day 21, fixed in 4 % paraformaldehyde and dehydrated sequentially in 20 and 30 % sucrose at 4 °C until they sank. The frozen glioma tissues were serially sectioned at a thickness of 12 μm, and the slide with the largest tumor area was stained with hematoxylin and eosin (H&E).

### Statistical analysis

The statistical analyses were performed using the GraphPad Prism 5 software package. All of the data are presented as the means ± SEM of three independent experiments. Comparisons of the mean values between the control and treated groups were performed using Student’s *t* test. A Kaplan-Meier survival curve and the log-rank test were used for the in vivo survival analysis. *P* values <0.05 were considered statistically significant.

## Results

### High *CRM1* expression predicts poor survival in patients with glioma

**Fig. 1:**
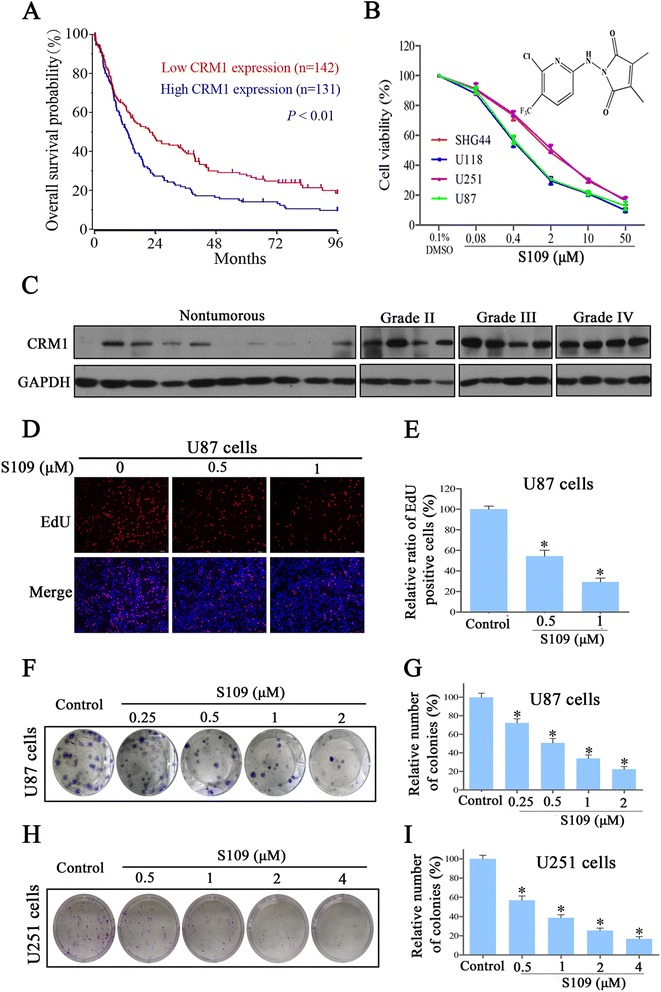
S109 inhibits the proliferation and colony formation ability of glioma cells. **a** Kaplan-Meier analysis of overall survival for the French data. CRM1 had high expression in 131 out of 273 cases of glioma was associated with poor patient survival. **b** Structure of S109 and assessment of cell viability. Cells were treated with vehicle or various concentrations of S109 for 72 h. The cell viability was measured using CCK-8 assays. **c** Total protein extracts isolated from non-tumorous brain tissues and glioma tissues were evaluated through western blotting assays. **d** Representative images from the EdU analysis of cell proliferation after treatment of U87 cells with S109. **f**, **h** S109 suppresses colony formation of U87 and U251 cells in a dose-dependent manner. **e**, **g**, **i** Quantitative results of the EdU incorporation and clonogenic assays of U87 and U251 cells

To evaluate the possibility that CRM1 is important for glioma, we analyzed the R2 genomics database, for which microarray-based gene expression and clinical outcome data were available. The prognosis analysis was conducted online, and cutoff values for separating high and low expression groups were determine by auto scan. As shown in Fig. 1a, *CRM1* gene was highly expressed in 131 out of 273 cases of glioma. The distinction between high and low *CRM1* was of prognostic significance, as the overall survival rate was markedly reduced in cases exhibiting high *CRM1* expression. Next, we assessed CRM1 protein expression in human glioma tissues through a western blot analysis and found that CRM1 was highly expressed in all tumor samples compared with non-tumorous brain tissues (Fig. 1c). We analyzed the R2 genomics database, for which microarray-based gene expression and clinical outcome data were available. These data indicate that CRM1 expression is significantly higher in grade III and IV gliomas than in grade II tumors (Additional file 1: Figure S1A). These findings indicated that up-regulation of *CRM1* in a subset of glioma leads to inferior outcome.

### S109 inhibits the proliferation and colony-formation ability of glioma cells

To examine the effect of S109 on glioma cell proliferation, we evaluated the viability of glioma cells treated with S109 using the CCK-8 and EdU assays. We found that S109 markedly inhibited cell proliferation in a dose-dependent manner in the five cell lines evaluated (Fig. 1b). Interestingly, the IC_50_ observed for the high-grade glioma cell lines U87 and U118 was twofold lower than that observed for the low-grade glioma cells lines U251 and SHG44. Furthermore, knockdown of CRM1 significantly decreased the growth of U87 cells (Additional file 1: Figure S1B and S1C). The EdU assay demonstrated that S109 significantly reduced the number of EdU-positive cells in a dose- (Fig. 1d) and time-dependent manner (Additional file 1: Figure S2). The exposure of U87 cells to 0.5 and 1 μM S109 reduced the proliferation of these cells by 54.2 and 29.3 %, respectively (Fig. 1e).

To evaluate the long-term effects of S109 on cell proliferation, a clonogenic assay was performed. As shown in Fig. 1f–i, S109 treatment induced a dose-dependent inhibition of the clonogenic potential of U87 and U251 cells. Compared with the control group, the colony formation in U87 cells was markedly decreased by 50.7, 34.1, and 22.2 % in response treatment with 0.5, 1, and 2 μM S109, respectively. Taken together, these results demonstrate that S109 can effectively inhibit the proliferation of glioma cells. More importantly, high-grade glioma cells are more sensitive to S109 treatment than low-grade glioma cells.

### S109 induces G1 arrest and modulates the expression of cell cycle regulators

**Fig. 2:**
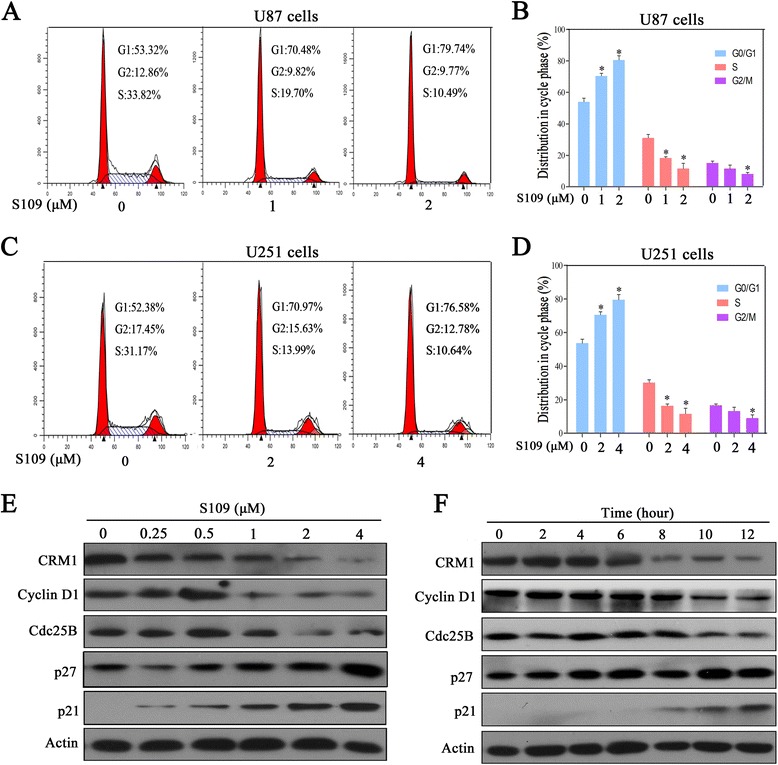
S109 induces cell-cycle arrest and regulates the expression of cell cycle-related proteins in glioma cells. **a**, **c** U87 and U251 cells were treated with S109 for 12 h. The cells were stained with PI and evaluated using a flow cytometer. **b**, **d** Quantitative analysis of the cell-cycle-phase distribution of the cells in the control group and the S109-treated group. **e**, **f** U87 cells were treated with 0.1 % DMSO or S109 for 12 h or treated with 2 μM S109 for different durations (0–12 h). The cells were then harvested and examined through western blot analysis with the indicated antibodies

To determine whether the S109-induced decrease in cell proliferation resulted from the abrogation of cell cycle progression, propidium iodide flow cytometry assays were performed. In the U87 cell population treated with the DMSO vehicle, 53.3 % of the cells were in the G1 phase, whereas the cell populations treated with 1 and 2 μM S109 exhibited higher percentages of cells (70.5 and 79.7 %, respectively) in the G1 phase (Fig. 2a, b). Similar effects on cell cycle progression were obtained in U251 cells treated with S109 (Fig. 2c, d). However, no significant changes in cell apoptosis were observed in either U87 or U251 cells treated with S109 (data not shown). These data clearly demonstrated that S109 selectively induces G1 cell-cycle arrest but does not affect apoptosis in glioma cells.

We next explored the effect of S109 on cell cycle regulation signaling through western blot analysis. As shown in Fig. 2e, f, S109 treatment significantly up-regulated the expression levels of the cell cycle inhibitory proteins p21 and p27 in a dose- and time-dependent manner. In addition, cyclin D1 and Cdc25B expression was markedly decreased in S109-treated cells compared with control cells. These results confirm that S109 induces G1 arrest by modulating multiple cell cycle regulatory proteins.

### S109 reversibly regulates the expression and function of CRM1 in human glioma cells

**Fig. 3:**
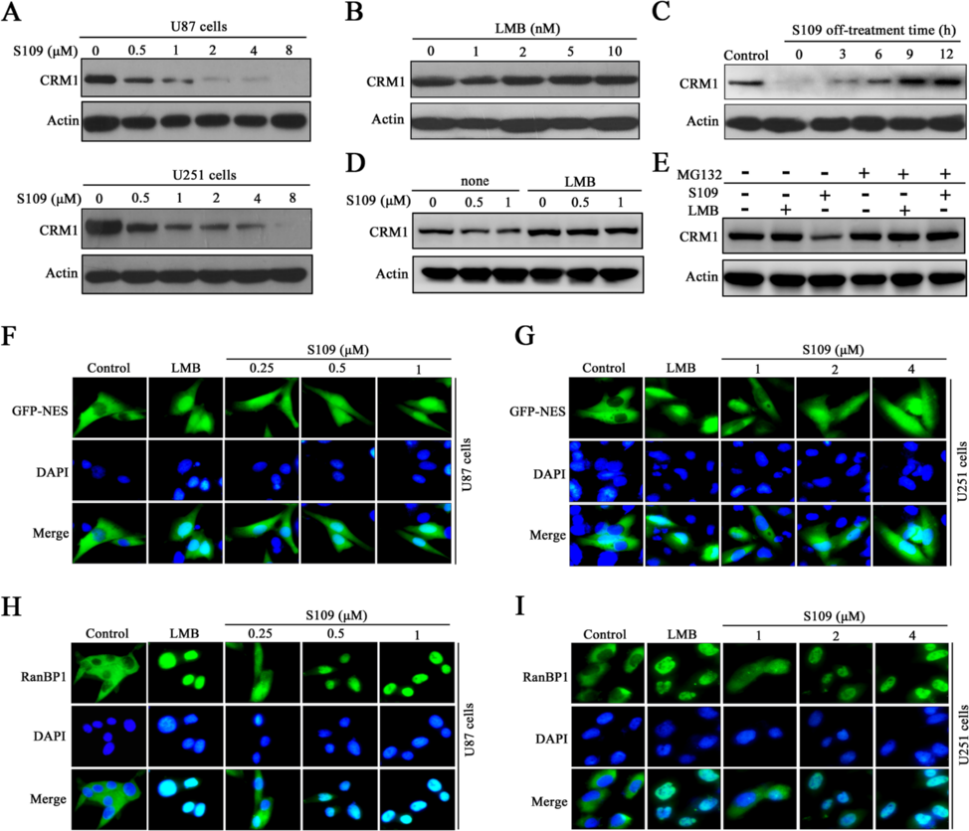
S109 decreases CRM1 protein expression and inhibits CRM1-dependent nuclear export in glioma cells. **a** U87 and U251 cells were incubated with S109 for 12 h, and the CRM1 protein levels were analyzed by immunoblotting. **b** U87 cells were treated with LMB for 12 h and then evaluated by western blot analyses. **c** U87 cells were treated with S109 (2 μM) for 12 h. The drugs were then washed out, and fresh medium was added. The cells were further incubated for the indicated times, and whole-cell lysates were analyzed by immunoblotting. **d** U87 cells were treated with S109 in the presence or absence of LMB (2 nM) for 12 h. The CRM1 protein levels were examined by western blot analysis. **e** U87 cells were treated with LMB (2 nM) and S109 (1 μM) in the presence or absence of MG132 (10 μM) for 12 h. Total proteins were extracted and used for immunoblotting analysis. **f**, **g** U87 and U251 cells were transiently transfected with the NES-GFP plasmid and treated with LMB and S109 at the indicated concentrations for 2 h. Fixed cells were stained with DAPI and analyzed by fluorescence microscopy. **h**, **i** U87 and U251 cells were incubated in the presence or absence of S109 for 2 h. The cells were then fixed, stained with RanBP1 antibody and DAPI, and analyzed by fluorescence microscopy

The effect of S109 on the level of CRM1 protein in glioma cells was investigated through western blot analysis. As shown in Fig. 3a, treatment with increasing concentrations of S109 significantly decreased the expression level of CRM1 protein in both U87 and U251 cells. However, the classic irreversible CRM1 inhibitor LMB did not induce degradation of CRM1 protein (Fig. 3b). Furthermore, pretreatment of U87 cells with LMB completely blocked S109-induced CRM1 protein degradation, suggesting that LMB and S109 compete for CRM1 binding (Fig. 3d). We then investigated the changes in the CRM1 protein level over time after cell transfer from S109-containing media to compound-free media. Six hours after the media exchange, the quantity of CRM1 protein in cells that had been treated with S109 was comparable to that of the control cells (Fig 3c). To determine whether S109-induced CRM1 degradation is associated with the ubiquitin-proteasome system, we treated U87 cells with S109 alone or in combination with the proteasome inhibitor MG132. S109 induced a rapid degradation of CRM1 protein in the absence of MG132, and this degradation was completely blocked by MG132-induced proteasome inhibition (Fig. 3e). These data suggest that S109 induces CRM1 protein degradation via the ubiquitin-proteasome system.

To investigate whether S109 is capable of functionally inactivating CRM1 in glioma cells, we analyzed the subcellular localization of NES-GFP and RanBP1, which are canonical markers of CRM1 inhibition. CRM1 exports cargo proteins via the recognition of a specific leucine-rich nuclear export signal (NES) consensus sequence in its substrates. In the control cells, NES-GFP was actively exported and localized predominantly to the cytoplasm (Fig. 3f, g). Notably, the exposure of U87 and U251 cells to S109 resulted in the progressive nuclear accumulation of NES-GFP. We then analyzed the subcellular localization of RanBP1, a protein known to be exported to the cytoplasm in a CRM1-dependent manner. As presented in Fig. 3h, i, RanBP1 localized exclusively to the cytoplasm in untreated glioma cells, but RanBP1 nuclear accumulation was observed as early as 2 h post-exposure to S109 in both U87 and U251 cells.

To examine whether S109 reversibly binds to CRM1 in glioma cells, we analyzed the subcellular localization of RanBP1 after cell transfer from S109-containing medium to S109-free medium. After 2 h of treatment, the S109-containing medium was removed, and the cells were washed twice and incubated with fresh medium for another 2 h. Interestingly, in the cells treated with S109, RanBP1 almost completely relocated to the cytoplasm after removal of the compound. However, in the cells incubated with LMB, RanBP1 was retained in the nucleus following transfer to medium without S109 (Additional file 1: Figure S3). Taken together, these results indicate that S109 can reversibly inhibit the function of CRM1 in glioma cells.

### S109 perturbs the core pathways associated with glioma by targeting CRM1-associated tumor-suppressor proteins

**Fig. 4:**
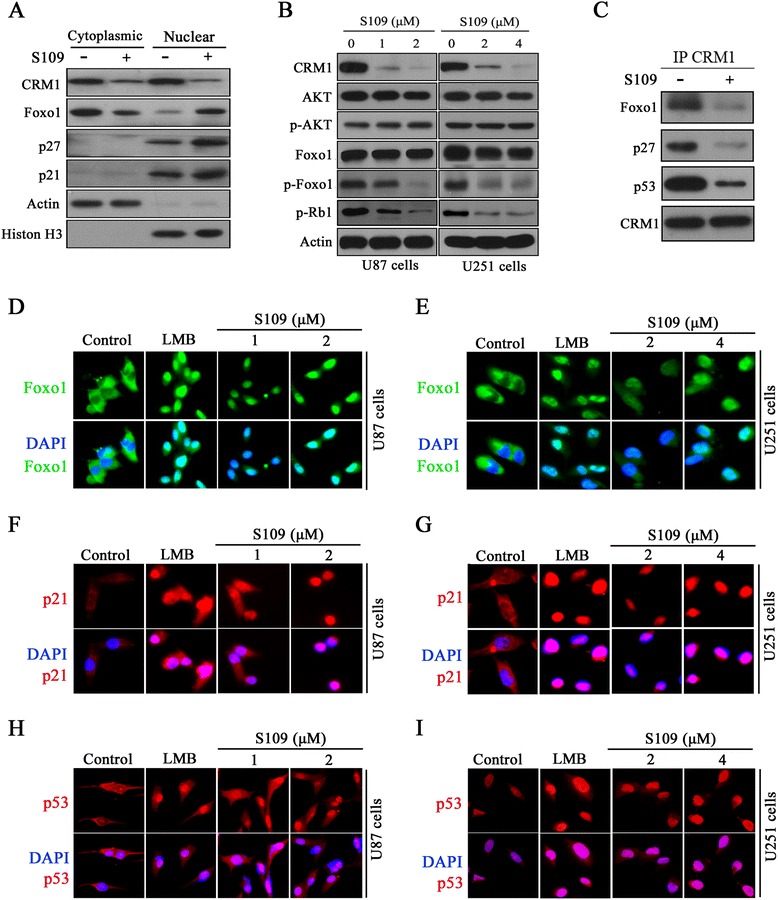
S109 perturbs the core pathways associated with glioma by promoting the nuclear retention of tumor-suppressor proteins. **a** U87 cells were treated with vehicle or S109 (2 μM) for 12 h. The cytoplasmic and nuclear protein extracts were analyzed by immunoblotting with the indicated antibodies. **b** U87 and U251 cells were cultured in the absence or presence of S109 for 24 h. Cell lysates were then prepared and analyzed by immunoblotting. **c** S109 disrupts the interaction of CRM1 with tumor-suppressor proteins. U87 cells were treated with S109 (2 μM) for 2 h. Cell lysates were immunoprecipitated using CRM1 antibody and evaluated by western blotting. **d**–**i** The cells were treated with vehicle, LMB (2 nM) or S109 at the indicated concentrations for 12 h. The localization of Foxo1, p21, and p53 was analyzed by fluorescence microscopy

To gain further insight into the mechanism underlying the cytotoxic effect of S109, we evaluated the nuclear localization of different tumor-suppressor proteins through western blot analysis and fluorescence microscopy. As presented in Fig. 4a, the exposure of U87 cells to S109 resulted in an increased nuclear accumulation of key tumor-suppressor proteins (Foxo1, p21, and p27) compared with the control. Foxo1 was detected exclusively in the cytoplasm in untreated cells, as determined by immunofluorescence (Fig. 4d, e). However, S109 treatment induced the nuclear accumulation of Foxo1 as early as 2 h post-treatment in both U87 and U251 cells. In addition, S109 also significantly enhanced the accumulation of p53 and p21 in the nucleus in both cell lines (Fig. 4f–i). The nuclear retention of these tumor-suppressor proteins is implicated in the triggering of cell-cycle arrest. These findings are consistent with the results of our cell-cycle analysis, suggesting that tumor-suppressor proteins play a role in CRM1-mediated cell-cycle arrest.

In glioma cells, Foxo1 and Rb1 can be inactivated by hyperphosphorylation [33, 34], and phosphorylated Foxo1 is exported from the nucleus in a CRM1-dependent manner. Therefore, we analyzed the effects of S109 on the phosphorylation of Foxo1 and Rb1 in glioma cells. A western blot analysis revealed a decrease in the level of the phosphorylated form of Foxo1 in glioma cells compared with the control cells (Fig. 4b). Similarly, S109 significantly decreased the level of phosphorylated Rb1 in a dose-dependent manner. However, no significant change in the levels of Akt phosphorylation was observed. Taken together, these data indicate that S109 might simultaneously perturb mediators of the critical pathways associated with glioma (the Foxo1, p53, and Rb1 signaling pathways) by promoting the nuclear retention of key tumor-suppressor proteins.

To determine whether S109 directly disrupts the interaction between CRM1 and cargo proteins, we incubated U87 cells in media with or without S109 and analyzed the protein extracts through immunoprecipitation assays. As shown in Fig. 4c, the tumor-suppressor proteins Foxo1, p27, and p53 were capable of binding CRM1 in the untreated cells. However, only low levels of these proteins co-precipitated with CRM1 in the treated samples, indicating that S109 treatment significantly inhibited the interaction of these proteins with CRM1. These results suggest that S109 might directly disrupt the interaction of CRM1 with the tumor-suppressor proteins Foxo1, p27, and p53.

### The CRM1 Cys528 mutation abrogates S109 activity in glioma cells

**Fig. 5:**
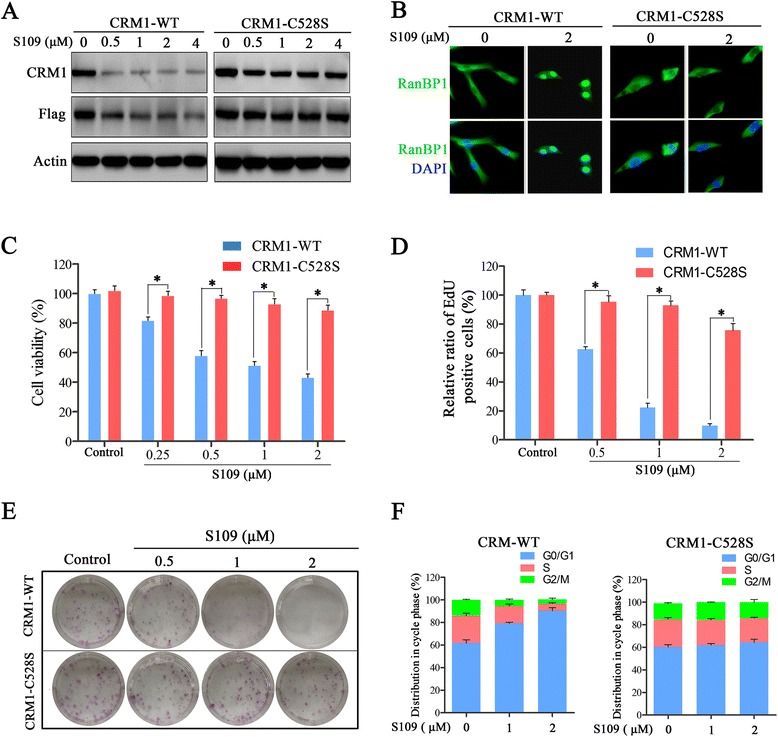
Mutation of CRM1 abolishes the anti-proliferative activity of S109 in glioma cells. **a** U87 cells expressing wild-type or C528S-mutant CRM1 were treated with the indicated doses of S109 for 12 h and evaluated by western blot analyses. **b** U87 cells expressing wild-type or C528S-mutant CRM1 were cultured in media with vehicle or S109 (2 μM) for 2 h. Fixed cells were stained for RanBP1 and DAPI and analyzed by fluorescence microscopy. **c** The cells were treated with vehicle or different concentrations of S109 for 72 h, and the cell viability was assessed through CCK-8 assays. **d** Quantitative analysis of the relative cell proliferation rate. The cell proliferation rate was measured using the EdU incorporation assay. **e** Assay of the colony formation ability of cells expressing wild-type or C528S-mutant CRM1. **f** Cell cycle distribution of U87 cells expressing wild-type or C528S-mutant CRM1 treated with S109 for 12 h

The Cys528 residue in the cargo-binding pocket of CRM1 is essential for the inhibitory effect of LMB [35]. Thus, we investigated the effects of the Cys528 mutation in CRM1 on S109 activity in glioma cells. We developed two U87 cell lines stably expressing either exogenous wild-type CRM1 or CRM1 with the Cys528 mutation. As shown in Fig. 5a, S109 induced CRM1 degradation in the cells expressing wild-type CRM1 but not in the cells expressing mutant CRM1. Furthermore, we did not observe RanBP1 nuclear accumulation in the cells expressing the CRM1 Cys528 mutant protein after S109 treatment (Fig. 5b).

We then explored the anti-tumor activity of S109 in U87 cells expressing mutant CRM1. Consistent with our previous observation, S109 treatment significantly inhibited the growth of the cells expressing wild-type CRM1 (Fig. 5c–e). However, the inhibitory effects of S109 on the growth of glioma cells were nearly abolished in the cells expressing Cys528 mutant CRM1. We also evaluated the effect of mutant CRM1 on the regulation of the cell cycle by S109. As shown in Fig. 5f, S109 treatment resulted in cell-cycle arrest at the G1 phase in the cells expressing wild-type CRM1. Notably, no marked changes in cell-cycle progression were observed after S109 treatment in the cells expressing mutant CRM1. Our data demonstrate that S109 can selectively bind the Cys528 residue of CRM1 with few off-target effects at the concentration used in these experiments.

### S109 treatment is effective and safe in an intracranial glioblastoma xenograft model

**Fig. 6:**
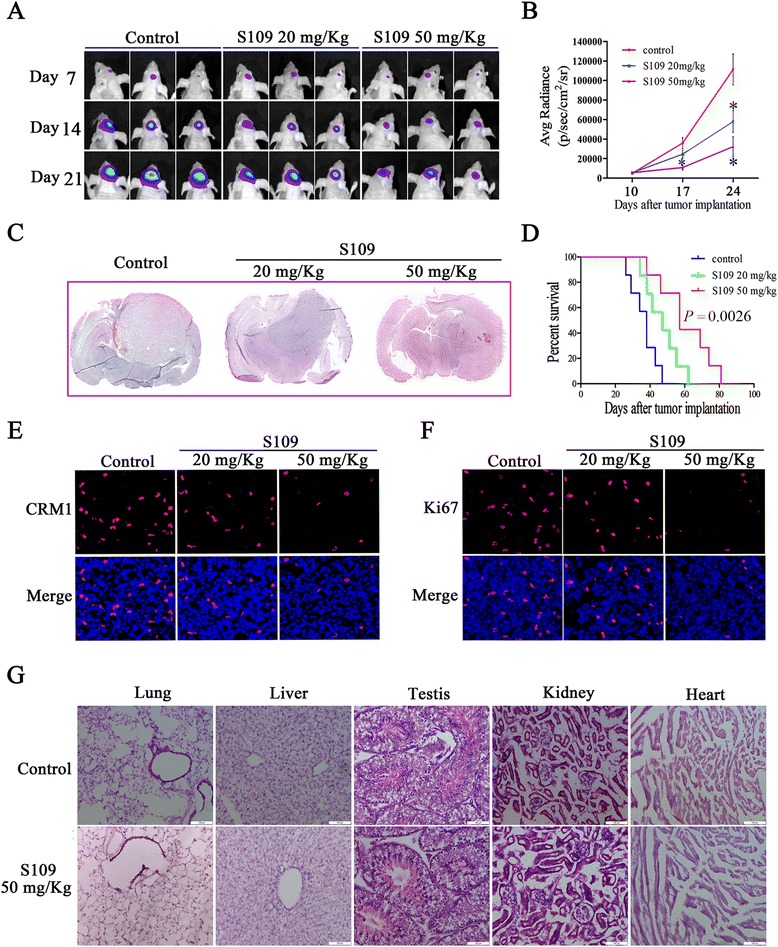
Effects of S109 in vivo. **a** S109 inhibits glioma cell growth in vivo. Animals with U87 xenografts were separated into the control and treatment groups once luciferase-expressing tumors were confirmed by a bioluminescence imaging system. Representative pseudocolor bioluminescence images obtained on days 7, 14, and 21 are shown. **b** Quantified luminescence signal obtained from the mice-bearing luciferase-labeled gliomas treated with S109. **c** Representative images of H&E staining of coronal sections from mouse brains with orthotopic tumors. **d** The survival of mice with tumors derived from the vehicle or S109-treated groups was measured by Kaplan-Meier survival curves. **e**, **f** Representative images of CRM1 and Ki-67 immunostaining of tumors dissected from control and S109-treated mice. **g** H&E-stained tissue sections of the lungs, livers, testes, kidneys, and hearts from the control and S109-treated mice were examined. *Scale bar*, 100 μm

To determine whether S109 exerts anti-tumor activity on glioblastoma cells in vivo, we evaluated its effect in an intracranial nude mouse model. S109 treatment was initiated at the first sign of tumor growth, as indicated through an imaging analysis. Treatment with S109 significantly suppressed the growth of U87 glioblastoma cells in vivo, as indicated by a marked increase in the bioluminescence signal observed in mice treated with the vehicle control compared with the S109-treated mice (Fig. 6a, b). In addition, 21 days after glioma transplantation, the transplanted gliomas in S109-treated mice were visibly smaller than those in the vehicle-treated mice (Fig. 6c). Consequently, the S109-treated mice exhibited significantly increased survival (Fig. 6d). Furthermore, our data demonstrated that S109 could effectively cross the blood-brain barrier (Additional file 1: Figure S4).

To examine the effect of S109 on CRM1 expression in vivo, we immunostained tumor tissues isolated from S109-treated mice. As expected, CRM1 protein expression was markedly decreased in the S109-treated tumor cells compared with the control tumor cells (Fig. 6e). In addition, we evaluated the proportion of Ki67-positive cells because this parameter is often used to evaluate tumor cell proliferation and histology. As shown in Fig. 6f, the proportion of Ki67-positive tumor cells in the mice treated with S109 was significantly lower than that found in the mice treated with vehicle. In addition, we evaluated the histology of cells from the liver, heart, testis, lungs, and kidneys of the vehicle- and S109-treated mice. The specimens were formalin-fixed and stained with hematoxylin/eosin, and no significant pathological changes were observed in the organs examined (Fig. 6g). These data indicate that S109 effectively targets intracranial gliomas without exerting any apparent toxicity to normal tissues.

## Discussion

Effective chemotherapies for gliomas are limited, and improving long-term survival in glioma patients is imperative [36]. Therefore, new modalities that can improve or replace the current treatments for gliomas are highly desirable. This study demonstrates that S109 exerts anti-tumor effects in glioma models in vitro and in vivo. Furthermore, we discovered that S109 exerts its anti-tumor effects by perturbing the three core pathways implicated in glioma: the RTK/AKT/Foxos signaling pathway and the p53 and Rb1 tumor-suppressor pathways.

CRM1 overexpression in human gliomas is associated with a poor prognosis and higher grade of malignancy [18]. Therefore, targeting CRM1 is a promising therapeutic strategy for gliomas. Our data support this hypothesis because we found that the CRM1 inhibitor S109 significantly suppresses the proliferation of glioma cells both in vitro and in vivo. The irreversible inhibitor of CRM1, KPT-330, also exerts anti-tumor activity in preclinical models of glioblastoma [37], but the molecular mechanism and cellular signaling pathways mediating the anti-glioma activity of KPT-330 remain unknown. The anti-glioblastoma effects of KPT-330 treatment result from the induction of apoptosis but are not associated with cell-cycle arrest [37]. In contrast, the anti-proliferative activity of S109 results from the induction of cell-cycle arrest in the G1 phase but does not appear to be associated with apoptosis. This discrepancy between the mechanism of KPT-330 and that of S109 might be related to the reversible binding of S109 to CRM1 or to a greater selectivity for CRM1. CRM1 can recognize and transport the leucine-rich nuclear export signal (NES) of cargo proteins, including transcription factors and tumor suppressor proteins such as p53, p21, p27, and Foxos. The inhibition of CRM1 function could disrupt and retain tumor suppressor proteins in the nucleus to induce cancer cell cycle arrest or apoptosis.

A number of genetic alterations have been implicated in processes that promote glioma progression, such as increased proliferation, resistance to apoptosis, and robust invasive capability [38]. The EGFR pathway has generated particular interest as a drug target. However, EGFR kinase inhibitors have demonstrated disappointing results in patients with gliomas [39]. The resistance of gliomas to this treatment approach might be due to the role of oncogenic mutations in genes that function up- and mid-stream of the EGFR/Akt pathway. This hypothesis raises the possibility that molecules that function upstream of the EGFR/Akt pathway might not be optimal therapy targets. Foxo transcription factors, the critical downstream transcription factors of the EGFR/Akt pathway, are inactivated by cytoplasmic mislocalization in glioma cells [40, 41]. High cytoplasmic Foxo1 expression in human gliomas is associated with a higher grade of malignancy [42]. However, it is difficult to develop direct inhibitors of transcription factors. In this manuscript, we report that the inhibition of CRM1 by S109 might significantly promote the nuclear retention of Foxo1, and our results indicate that redirecting Foxo1 to the nucleus by inhibiting CRM1 might be an effective approach for treating gliomas.

Inactivation of the p53 and Rb1 tumor-suppressor pathways is frequently observed in glioma cells. The proteins p14/ARF and p16/INK4A are the key regulators of the p53 and Rb1 signaling pathways, respectively [43]. In human gliomas, one of the most common genetic aberrations is the homozygous deletion of *CDKN2A*. Because *CDKN2A* encodes both p14/ARF and p16/INK4A, the deletion of *CDKN2A* results in the disruption of both the p53 and Rb1 pathways [4]. We found that S109 treatment induces the nuclear accumulation of p53 and decreases the level of Rb1 phosphorylation. Rb1 is a well-characterized protein that functions as a key negative regulatory protein of cell-cycle progression from the G1 phase. Thus, one of the mechanisms through which S109 induces G1 cell-cycle arrest is by promoting the nuclear retention of Rb1. Interestingly, we observed that high-grade glioma cells are more sensitive to S109 treatment than low-grade cells. This difference might be related to the observation that the three core oncogenic pathways are more frequently disrupted in high-grade glioma cells [4]. Taken together, our results indicate that S109 treatment can simultaneously target the three core pathways implicated in glioma. Therefore, the targeted inhibition of CRM1 is an attractive strategy for the treatment of gliomas.

## Conclusions

In summary, our results affirm that S109 is an effective reversible inhibitor of CRM1 and that CRM1 is an attractive molecular target for the development of molecular therapeutics for glioma. In particular, we have provided strong evidence that the underlying mechanism of action of S109 involves a perturbation of the three core pathways in human glioma cells that is mediated by the nuclear retention of tumor-suppressor proteins. These findings highlight the potential of future clinical trials evaluating the therapeutic potential of S109 for the treatment of human gliomas.

## Additional file

Additional file 1: Figure S1 Knockdown of CRM1 inhibits the proliferation of glioma cells. (A) Expression of CRM1 was analyzed from 273 samples of different glioma types based on R2 genomics database. (B) U87 cells were infected with either a CRM1-specific shRNA or control shRNA. CRM1 knockdown was evaluated by immunoblotting. (C) The proliferation of U87 cells was measured by CCK-8 assay. Figure S2. S109 suppresses glioma cell proliferation in a time-independent manner. (A and B) U87 and U251 cells were treated with S109 at the indicated doses for different periods of time. Cell proliferation was assessed using the EdU incorporation assay. Figure S3. S109 reversibly inhibits the function of CRM1. (A and C) U87 and U251 cells were incubated with S109 or LMB for 2 h. Fixed cells were stained for RanBP1 and DAPI and analyzed by fluorescence microscopy. (B and D) U87 and U251 cells were treated with S109 or LMB for 2 h. Next, the inhibitors were washed out and fresh medium was added. The cells were further incubated for 2 h, stained with anti-RanBP1 and DAPI, and analyzed by fluorescence microscopy. Figure S4. The pharmacokinetic characteristics of S109 in plasma and cerebrospinal fluid (CSF). S109 (50 mg/Kg) was administered to rats via intraperitoneal injections. Blood and cerebrospinal fluid samples were collected at 10, 20, and 30 min post-dose. The concentrations of S109 in plasma and cerebrospinal fluid were determined by LC-MS/MS, respectively. (DOCX 5829 kb)

## Abbreviations

CRM1: Chromosomal region maintenance 1
EGFR: Epidermal growth factor receptor
LMB: Leptomycin B
NES: Nuclear export signal
RTK: Receptor tyrosine kinases
XPO1: Exportin 1
