# Supplementary material for: CRM1/XPO1 is associated with clinical outcome in glioma and represents a therapeutic target by perturbing multiple core pathways
Source: J Hematol Oncol. 2016 Oct 12;9:108. doi: 10.1186/s13045-016-0338-2 (PMC5059893; doi:10.1186/s13045-016-0338-2)
Supplement: Additional file 1: Figure S1 — Knockdown of CRM1 inhibits the proliferation of glioma cells. (A) Expression of CRM1 was analyzed from 273 samples of different glioma types based on R2 genomics database. (B) U87 cells were infected with either a CRM1-specific shRNA or control shRNA. CRM1 knockdown was evaluated by immunoblotting. (C) The proliferation of U87 cells was measured by CCK-8 assay. Figure S2. S109 suppresses glioma cell proliferation in a time-independent manner. (A and B) U87 and U251 cells were treated with S109 at the indicated doses for different periods of time. Cell proliferation was assessed using the EdU incorporation assay. Figure S3. S109 reversibly inhibits the function of CRM1. (A and C) U87 and U251 cells were incubated with S109 or LMB for 2 h. Fixed cells were stained for RanBP1 and DAPI and analyzed by fluorescence microscopy. (B and D) U87 and U251 cells were treated with S109 or LMB for 2 h. Next, the inhibitors were washed out and fresh medium was added. The cells were further incubated for 2 h, stained with anti-RanBP1 and DAPI, and analyzed by fluorescence microscopy. Figure S4. The pharmacokinetic characteristics of S109 in plasma and cerebrospinal fluid (CSF). S109 (50 mg/Kg) was administered to rats via intraperitoneal injections. Blood and cerebrospinal fluid samples were collected at 10, 20, and 30 min post-dose. The concentrations of S109 in plasma and cerebrospinal fluid were determined by LC-MS/MS, respectively. (DOCX 5829 kb) [file 13045_2016_338_MOESM1_ESM.docx]

**Additional file 1**


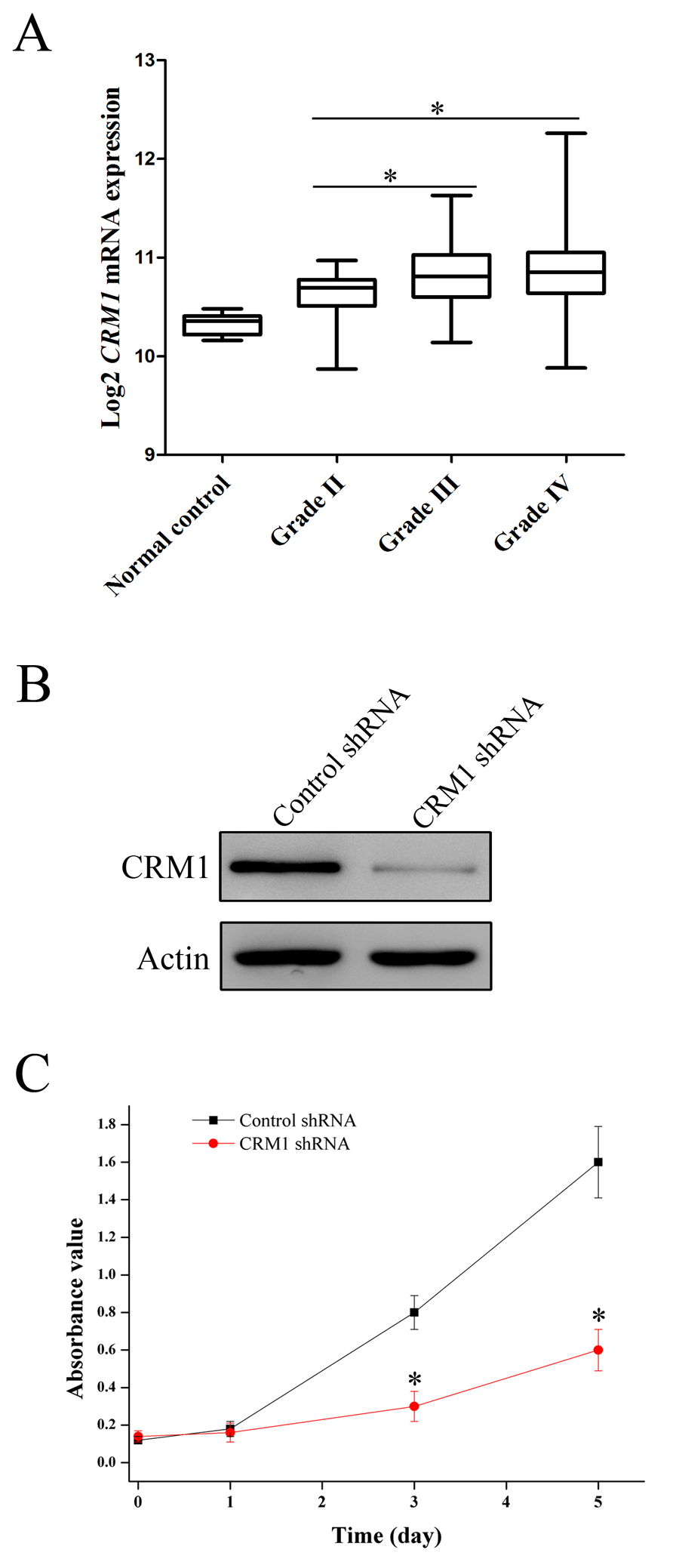


**Figure S1. Knockdown of CRM1 inhibits the proliferation of glioma cells.** (A) Expression of CRM1 was analyzed from 273 samples of different glioma types based on R2 genomics database. (B) U87 cells were infected with either a CRM1 specific shRNA or control shRNA. CRM1 knockdown was evaluated by immunoblotting. (C) The proliferation of U87 cells was measured by CCK-8 assay.


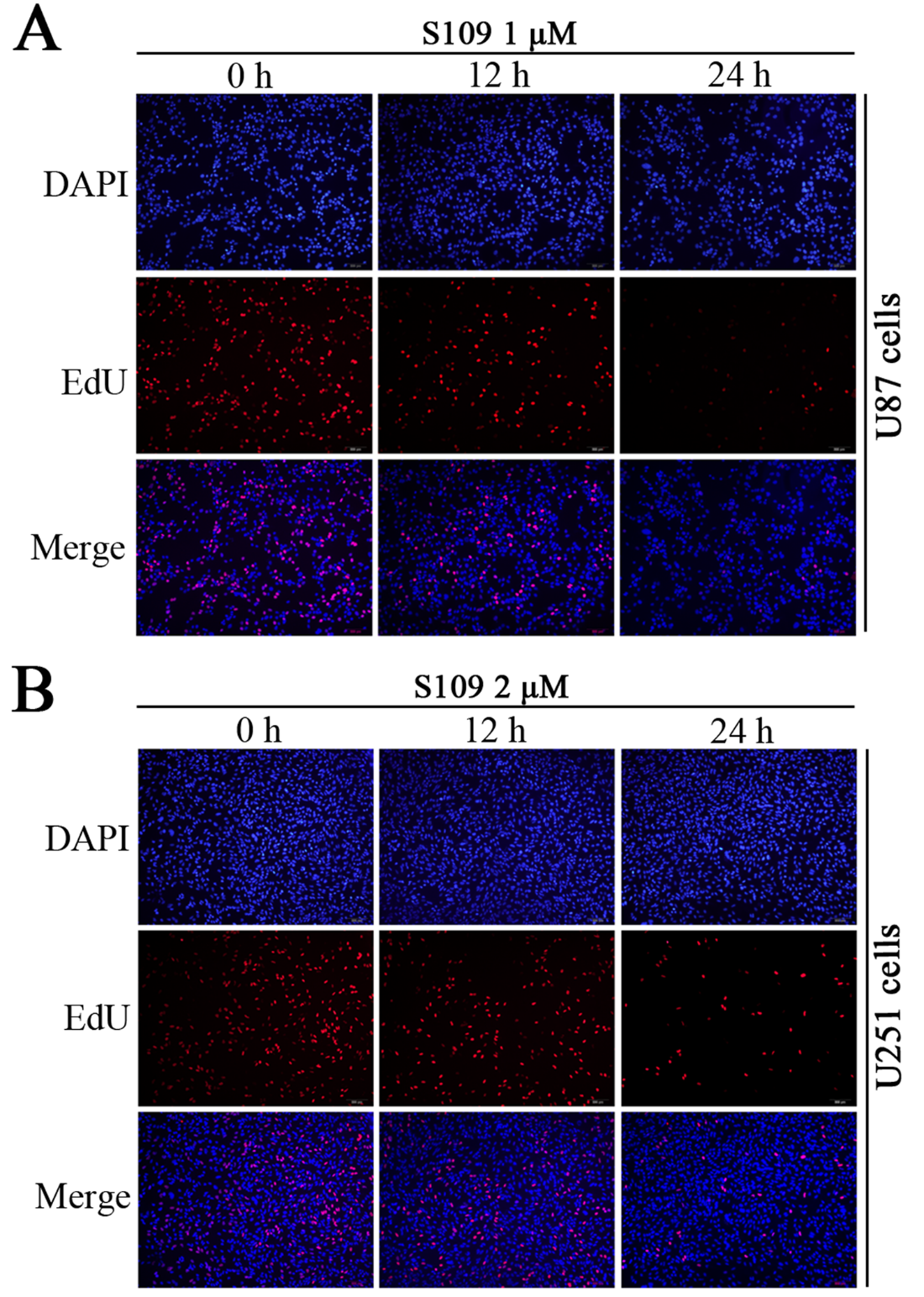


**Figure S2. S109 suppresses glioma cell proliferation in a time-independent manner.** (**A** and **B**) U87 and U251 cells were treated with S109 at the indicated doses for different periods of time. Cell proliferation was assessed using the EdU incorporation assay.


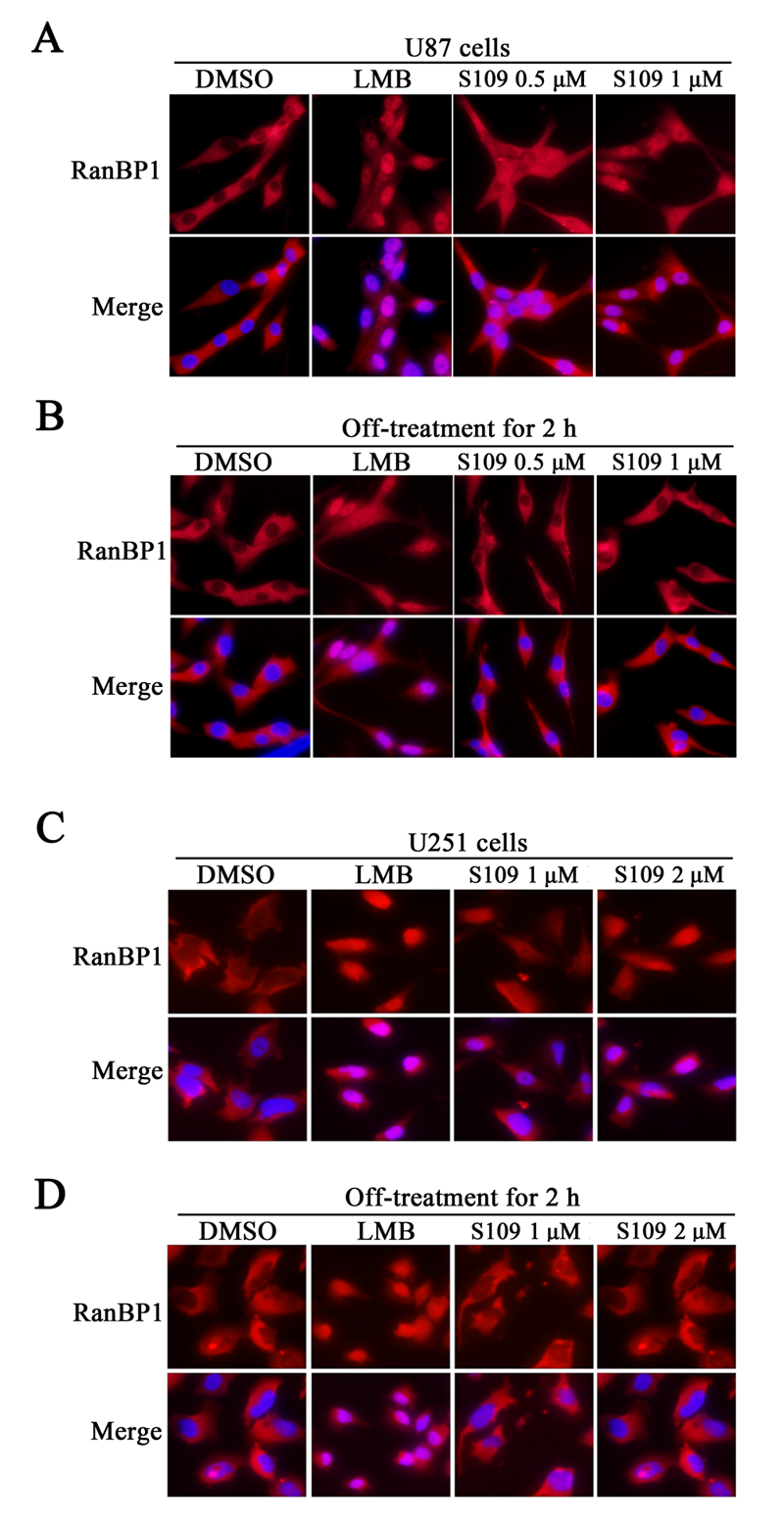


**Figure S3. S109 reversibly inhibits the function of CRM1.** (**A** and **C**) U87 and U251 cells were incubated with S109 or LMB for 2 h. Fixed cells were stained for RanBP1 and DAPI and analyzed by fluorescence microscopy. (**B** and **D**) U87 and U251 cells were treated with S109 or LMB for 2 h. The inhibitors were then washed out, and fresh medium was added. The cells were further incubated for 2 h, stained with anti-RanBP1 and DAPI and analyzed by fluorescence microscopy.





**Figure S4. The pharmacokinetic characteristics of S109 in plasma and cerebrospinal fluid (CSF).** S109 (50 mg/Kg) was administered to rats via intraperitoneal injections. Blood and cerebrospinal fluid samples were collected at 10, 20 and 30 min postdose. The concentrations of S109 in plasma and cerebrospinal fluid were determined by LC-MS/MS, respectively.
